# Differential regulation of BACE1 expression by oxidative and nitrosative signals

**DOI:** 10.1186/1750-1326-6-17

**Published:** 2011-03-03

**Authors:** Young-Don Kwak, Ruishan Wang, Jing Jing Li, Yun-Wu Zhang, Huaxi Xu, Francesca-Fang Liao

**Affiliations:** 1 Department of Pharmacology, University of Tennessee Health Science Center, College of Medicine, 874 Union Avenue, Memphis TN, 38163, USA; 2 Institute for Biomedical Research and Fujian Provincial Key Laboratory of Neurodegenerative Disease and Aging Research, Xiamen University, Xiamen 361005, China; 3 Del E. Webb Center for Neuroscience, Aging, and Stem Cell Research, Sanford-Burnham Medical Research Institute, 10190 North Torrey Pines Road, La Jolla, CA 92037, USA

## Abstract

**Background:**

It is well established that both cerebral hypoperfusion/stroke and type 2 diabetes are risk factors for Alzheimer's disease (AD). Recently, the molecular link between ischemia/hypoxia and amyloid precursor protein (APP) processing has begun to be established. However, the role of the key common denominator, namely nitric oxide (NO), in AD is largely unknown. In this study, we investigated redox regulation of BACE1, the rate-limiting enzyme responsible for the β-cleavage of APP to Aβ peptides.

**Results:**

Herein, we studied events such as S-nitrosylation, a covalent modification of cysteine residues by NO, and H_2_O_2_-mediated oxidation. We found that NO and H_2_O_2 _differentially modulate BACE1 expression and enzymatic activity: NO at low concentrations (<100 nM) suppresses BACE1 transcription as well as its enzymatic activity while at higher levels (0.1-100 μM) NO induces S-nitrosylation of BACE1 which inactivates the enzyme without altering its expression. Moreover, the suppressive effect on BACE1 transcription is mediated by the NO/cGMP-PKG signaling, likely through activated PGC-1α. H_2_O_2 _(1-10 μM) induces BACE1 expression via transcriptional activation, resulting in increased enzymatic activity. The differential effects of NO and H_2_O_2 _on BACE1 expression and activity are also reflected in their opposing effects on Aβ generation in cultured neurons in a dose-dependent manner. Furthermore, we found that BACE1 is highly S-nitrosylated in normal aging brains while S-nitrosylation is markedly reduced in AD brains.

**Conclusion:**

This study demonstrates for the first time that BACE1 is highly modified by NO via multiple mechanisms: low and high levels of NO suppress BACE1 via transcriptional and post translational regulation, in contrast with the upregulation of BACE1 by H_2_O_2_-mediated oxidation. These novel NO-mediated regulatory mechanisms likely protect BACE1 from being further oxidized by excessive oxidative stress, as from H_2_O_2 _and peroxynitrite which are known to upregulate BACE1 and activate the enzyme, resulting in excessive cleavage of APP and Aβ generation; they likely represent the crucial house-keeping mechanism for BACE1 expression/activation under physiological conditions.

## Background

It is well established that both cerebral hypoperfusion/stroke and type 2 diabetes are risk factors for Alzheimer's disease (AD) [1-4]. Oxidative and nitrosative stresses are common denominators for these age-related diseases [5]. Oxidative stress is associated with β-amyloid peptide (Aβ) accumulation in the brains of AD patients [6,7]. Aβ is generated by sequential proteolytic cleavages of the transmembrane amyloid precursor protein (APP) by two membrane-bound proteases, β-secretase (BACE1) and the γ-secretase complex composed of presenilin 1 (PS1), nicastrin, APH-1 and PEN-2 [8-10]. In AD brains, the specific regions affected by Aβ deposition correlate with increased BACE1 protein levels and activity [11-15]. Together with the observation that amyloid pathology was diminished in mice deficient in BACE1 [16,17], these findings strongly suggest that BACE1 elevation leads to enhanced Aβ production and deposition in AD. Given the central role of Aβ in AD pathogenesis and the fact that BACE1 is the rate-limiting enzyme in APP processing and Aβ generation, BACE1 remains one of the most important therapeutic targets for treating AD.

Compelling evidence indicates that BACE1 expression is tightly regulated at both the transcriptional and translational levels [18,19]. A number of transcriptional factors have been identified that positively or negatively regulate BACE1 gene expression under both basal and cell-stressed conditions, such as inflammation [20-22]. Some studies suggest that BACE1 is regulated by specific microRNAs, post-transcriptionally [23-25]. In addition to inflammation, other conditions have also been shown capable of causing increased BACE1 expression in the brain, including oxidative stress, traumatic brain injury [26], hypoxia and ischemia [27,28]. Neuronal cells exposed to oxidizing agents such as H_2_O_2 _and 4-HNE (4-hydroxynonenal) also show increased BACE1 expression [29,30]. Moreover, injury and stress-induced increases in lipid peroxidation were recently demonstrated to be responsible for upregulation of BACE1 expression in the brain of a genetic mouse model [31]. While the molecular mechanism underlying ischemia/hypoxia-induced BACE1 activation and APP processing has been extensively studied [32-34], the molecular basis of oxidative/nitrosative signal-mediated BACE1 regulation is virtually unknown.

The diffusible gaseous nitric oxide (NO) molecule is generated by activated nitric oxide synthase (NOS) which exists in at least three isoforms (neuronal nNOS, inducible iNOS and endothelial eNOS). NO is known to have pleiotropic physiological and pathological effects depending on the target tissue and cell type [35,36]. For instance, in blood vessels NO functions as a vasodilator, while in the nervous system NO acts as a neurotransmitter. However, if produced in excess and in the appropriate redox state, NO can be neurotoxic. It is well-accepted that NO released from eNOS is protective by promoting vasodilation while NO produced from overactivation of nNOS or iNOS under inflammatory conditions (which generates over 1000-fold more NO compared to constitutive nNOS and eNOS) is deleterious [37,38]. Often, the damage caused by excessive amounts of NO is affected through a neurotoxic derivative named peroxynitrite formed by combining with superoxide anions (O_2_). Signaling by NO is transduced mainly by targeted modifications of critical cysteine residues in proteins, including S-nitrosylation and S-oxidation, as well as by lipid and tyrosine nitration [39,40]. S-nitrosylation, the covalent modification of a thiol group by NO, probably represents the major mechanism of NO signaling [41-44]; it plays a critical role in fine-tuning a number of important molecules in the CNS related to cell death, protein folding and degradation [45,46]. We recently demonstrated that the PI3K/Akt signaling pathway, which is arguably the most important pro-survival pathway in neurons, is sensitive to this redox regulation; S-nitrosylation and NO-mediated regulation of PTEN represents a novel and crucial mechanism to activate PI3K/Akt signaling [47].

In this study, we investigated NO-mediated regulation of BACE1 and compared it to that of H_2_O_2_. We speculated that the dual functions of NO may exert differential effects on BACE1 regulation. Indeed, we observed different effects from NO at low and high levels and found that the two conditions also regulate BACE1 differently from H_2_O_2_-mediated oxidation; these may represent the actual redox regulation of BACE1 during the various stages of AD pathogenesis.

## Results

### Exogenous and endogenous NO donors induce S-nitrosylation of BACE1 in cultured neurons

**Figure 1:**
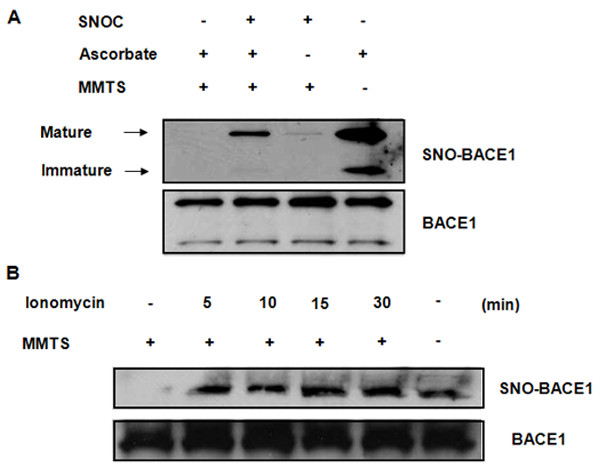
**Exogenous and endogenous NO donors induce S-nitrosylation of BACE1 in cultured PRCN neurons**. (A) Nitrosylation of BACE1 by SNOC was detected with biotin-switch assays: After exposure to SNOC (100 μM) for 30 min, to detect S-nitrosylated cystein residues, the free cysteine residues of BACE1 were first masked by methylthiolation with MMTS. Nitrosothiols were then selectively reduced by ascorbate to reform free thiol groups, which reacted with biotin-HPDH. Subsequently, biotin-HPDH conjugated proteins can be precipitated by streptavidin beads. In this experiment, MMTS was not added, to serve as a positive control, since all the cysteine residues in BACE1 can react with biotin-HPDP (last lane). Samples not treated with ascorbate (third lane) were used as a negative control due to the lack of reactive cysteine residues to biotin-HPDH. (B) Effect of Ca^2++ ^ionophore, ionomycin (1 μM), on SNO-BACE1 was examined at various time points (0, 5, 10, 15, and 30 min) by biotin-switch assay.

Using biotin-switch assays [48], we found that S-nitrosylation of BACE1 (SNO-BACE1) can be rapidly induced in primary cultured cortical neurons treated with the physiological NO donor S-nitrosocysteine (SNOC) in a dose-dependent manner. Detectable nitrosylation was induced by 100 nM SNOC with a plateau seen at 100 μM of SNOC. Notably, specific nitrosylation occurred only on the mature form of BACE1 (upper band, Figure 1A). We therefore only present data for the mature BACE1 in most figures and Western blot analyses. This was also confirmed by a more quantitative fluorescent assay (DAN assay, [49]) using purified recombinant BACE1 (data not shown). Additional neurotoxic compounds that induce NO generation, such as ionomycin, also induced robust SNO-BACE1 within minutes (Figure 1B) which lasted for several hours, even after the NO donors were removed from the cultured media. Furthermore, we found that the induced BACE1 nitrosylation was diminished by DTT treatment (1 mM, data not shown).

### SNOC displays differential effects on BACE1 protein levels at low and high concentrations

**Figure 2:**
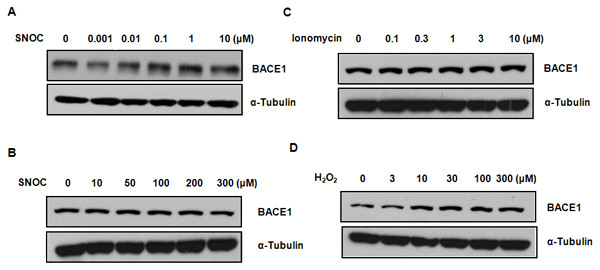
**Effect of nitrosative and oxidative stresses on BACE1 protein levels**. To examine differential effects of nitrosative and oxidative stresses on BACE1 protein levels, various concentrations of SNOC (0, 0.001, 0.01, 0.1, 1, 10, 50, 100, 200, and 300 μM) (A and B), Ionomycin (0, 0.1, 0.3, 1, 3, 10 μM) (C) and H_2_O_2 _(0, 3, 10, 30, 100, 300 μM) (D) were added to primary rat cortical neurons for 1 hr. Then, BACE1 protein level was measured by probing BACE1 with a specific monoclonal antibody (5D3). While lower concentrations of SNOC downregulated the BACE1 protein level, high concentrations of SNOC showed no effect on BACE1 protein levels (A and B). BACE1 expression level was not affected by endogenous NO induced by ionomycin (C). However, lower concentration of H_2_O_2 _significantly upregulated BACE1 protein levels (D).

We then examined the effects of SNOC on BACE1 protein levels and surprisingly found that SNOC exerts different effects at low and high concentrations. Specifically, SNOC at lower than 100 nM reduced BACE1 expression by ~ 50% (Figure 2A) which was restored to normal basal levels at SNOC concentrations higher than 100 nM. Interestingly, SNOC at concentrations higher than 100 nM exerted no effect on BACE1 protein expression levels (Figure 2B), The endogenous NO induced by ionomycin showed no effect on BACE1 at various concentrations (Figure 2C). In contrast, H_2_O_2 _induced BACE1 expression at concentrations as low as 10 μM in a dose-dependent manner (Figure 2D).

### Low NO levels suppress BACE1 transcription which is likely mediated through cGMP-PKG-upregulated PGC-1alpha

**Figure 3:**
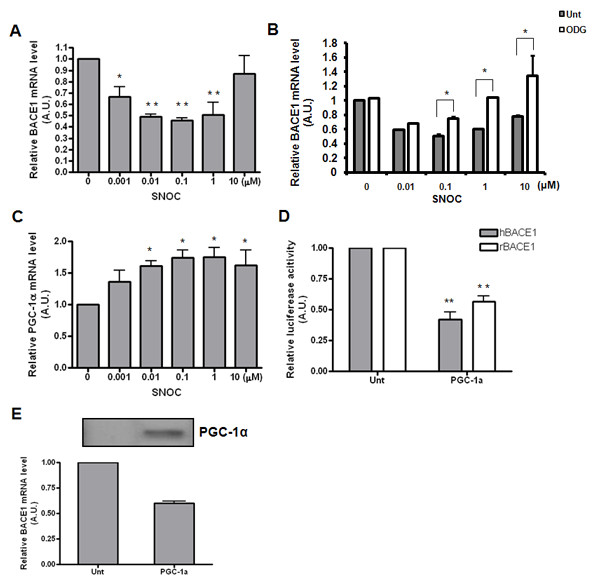
**Low NO concentrations suppress BACE1 transcription, which is likely mediated through cGMP-PKG-upregulated PGC-1α**. (A) Effect of nitrosative stress on BACE1 mRNA levels was examined by qRT-PCR. Lower concentrations of SNOC inhibited while higher concentrations of SNOC had no effect on BACE1 transcription. (B) Effect of cGMP inhibitor, 1H-[1.2.4]Oxadiazolo[4,3-a]quinoxalin-1-one (ODG, Sigma) in BACE1 transcription. (C) Effect of NO on PGC-1α mRNA levels. (D) Effect of PGC-1α overexpression on the promoter activity BACE1 from both human and rat origins, as assessed by luciferase assay with/without transfection of PGC-1α. (E) Effect of PGC-1α on BACE1 mRNA levels was assessed with qRT-PCR.

It was previously shown that oxidative stressors such as H_2_O_2 _can upregulate BACE1 at the transcriptional level [50,51]. To investigate the possibility that the low-concentration NO-mediated BACE1 suppression is effected through transcriptional inhibition, we conducted quantitative RT-PCR on endogenous BACE1 in rat primary neurons treated with various concentrations of SNOC. We found that the BACE1 messages were reduced in a dose-dependent manner from 0.1-100 nM and then slowly restored from 1-100 μM to their basal levels (Figure 3A). The maximum reduction of BACE1 messages was 2-fold, seen between 10-100 nM, which was consistent with the 50% reduction in BACE1 protein levels. We therefore believe that the low-concentration NO-mediated BACE1 suppression is mainly effected through transcriptional regulation.

Since NO signaling at low NO concentration is usually mediated by the classic cGMP activated PKG pathway, we then tested the effect of a potent NO/cGMP-PKG inhibitor, 1H-[1.2.4]Oxadiazolo[4,3-a]quinoxalin-1-one (ODG), and showed that it abolished the suppressive effect of low NO on BACE1 transcription (Figure 3B). Furthermore, we tested the potential role of the coactivator of PPARγ, named PGC-1α, which is known to be upregulated by low-concentration NO-cGMP signals [52]. In parallel with BACE1 transcription, we examined the mRNA levels of PGC-1α under various SNOC concentrations and found an inverse correlation between the mRNA levels of BACE1 and PGC-1α (Figure 3C). Because PPARγ has been suggested to suppress BACE1 via its responsive element (PPRE, [53]), it is possible that the upregulated PGC-1α is involved in BACE1 transcriptional control. Indeed, we show here that overexpression of PGC-1α can downregulate BACE1 transcription, measured by its effect on endogenous BACE1 message levels (Figure 3D); a similar effect was also seen on the transfected rat BACE1 promoter using a luciferase assay (Figure 3E). Taken together, these results strongly suggest that PGC-1α may be the major factor mediating the BACE1 suppression in response to cGMP-PKG signaling.

### SNOC and H_2_O_2 _have opposing effects on BACE1 enzymatic activity and subsequent Aβ generation

**Figure 4:**
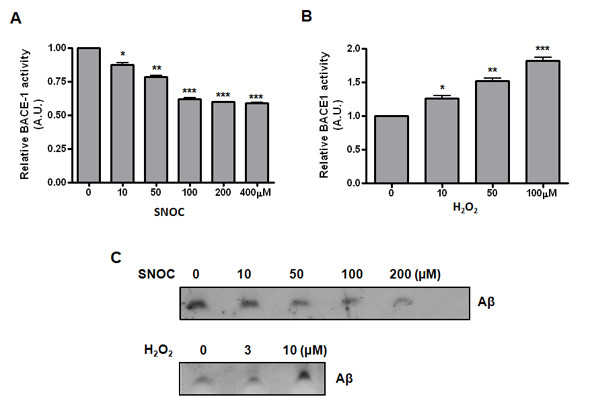
**SNOC and H**_**2**_**O**_**2 **_**have opposing effects on BACE1 enzymatic activity and subsequent Aβ generation**. (A and B) Effect of nitrosative and oxidative stresses on BACE1 activity was measured by a fluorescent based β-secretase activity assay kit (Sigma). Different doses of SNOC and H_2_O_2 _were added to N2a-695 stable cells expressing human APP695 proteins, for 1 hr. Subsequently, cells were lysed by lysis buffer for measurement of BACE1 activity. (C) For further investigation of the effect of SNOC and H_2_O_2 _on Aβ generation, conditioned media was collected after 4 hrs incubation with OPTI-MEM media from cells treated with various concentrations of SNOC and H_2_O_2 _for 1 hr. Then, secreted Aβ peptides were immunoprecipitated with 6E10 antibody and protein G/A beads to measure secreted Aβ peptides.

Since S-nitrosylation occurs at critical Cys residues which often leads to alteration of the structure and function of the target proteins, we examined BACE1 enzymatic activity upon SNOC and H_2_O_2 _treatments. Interestingly, SNOC-BACE1 shows reduced enzymatic activity while oxidized BACE1 exhibits enhanced activity; assays were performed with a commercial kit using both cell lysates and purified recombinant protein (Figure 4A). Accordingly, SNOC and H_2_O_2 _display opposite effects on Aβ generation, as determined by IP-Westerns using an antibody specific to Aβ (6E10). Consistent with the dose-dependent effects of H_2_O_2 _on BACE1 transcription, H_2_O_2 _also shows an effect dependent on the same dosages that induce BACE1 and Aβ generation, with a maximum effect seen at 10 μM (Figure 4B).

### SNO-BACE1 levels decrease in late stage AD brains which inversely correlates with BACE1 protein levels

**Figure 5:**
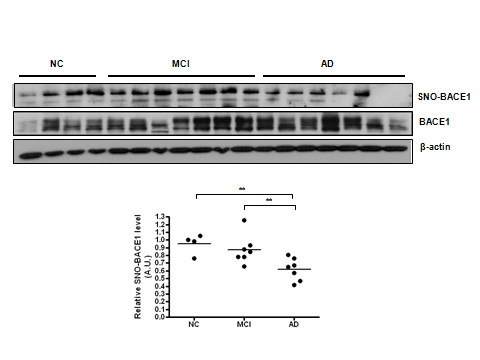
**SNO-BACE1 levels decrease in late stage AD brains which inversely correlates with BACE1 protein levels**. (A) SNO-BACE1 levels were detected by immunoblot analysis following biotin-switch assays in NC, MCI, and AD patient brain samples. Total BACE1 levels were detected by Western blot analysis. (B) Quantification of the Western blots using densitometry analysis reveals a statistically significant elevation of SNO-BACE1 levels in AD brains compared to NC and MCI brains ** indicates *P *< 0.005.

We next investigated whether S-nitrosylation of BACE1 occurred *in vivo *in neurodegenerative disorders associated with high levels of nitrosative stress, such as stroke and AD. We included in our tests those specimens taken from autopsy patients diagnosed at an early stage of AD called mild cognitive impairment (MCI) and compared them to age-matched control brain specimens (i.e., patients died from disorders not related to CNS). We chose to examine the entorhinal cortices, the most vulnerable region in AD brains. The patient cohort and information are summarized in the additional table. From semi-quantitative profiling of SNO-BACE1 and total BACE1 in 18 human brains, we found that SNO-BACE1 was at high levels in control and MCI samples and was markedly reduced in the late stage AD brains (Figure 5).

## Discussion

It is widely accepted that oxidative stress is one of the earliest changes that occurs in the pathogenesis of AD, arising from the imbalance between increased production of reactive oxygen and nitrogen species and impaired antioxidant defenses, as reflected in the accumulation of oxidative damage to macromolecules detected in MCI and AD brains [39,40]. In this work, we present *in vitro *and *in vivo *evidence of NO-mediated regulation of BACE1. We are the first to demonstrate that NO, at different levels, can exert differential regulation on BACE1: at low levels, NO suppresses BACE1 transcription while at modest to high levels, NO induces S-nitrosylation of BACE1 and inactivates the enzyme. Furthermore, we show that S-nitrosylation of BACE1 occurs in normal aging and MCI brains but is significantly diminished in late stage AD brains. Given the central role of Aβ in AD pathogenesis and the fact that BACE1 is the rate-limiting enzyme in APP processing and Aβ generation, the redox regulation of BACE1 identified herein may represent a novel and crucial mechanism for keeping BACE1 at physiological levels/activity.

The multifaceted actions of the NO group can be classified into two categories: classic NO-mediated/cGMP-dependent actions and reactive nitrogen species-mediated/cGMP-independent actions. The cGMP-dependent actions often play critical roles in a variety of physiological processes, including NO-mediated vasodilation. In contrast, cGMP-independent actions are more frequently postulated to be involved in the pathological responses which are primarily effected by nitrosative post-translational modifications of proteins such as S-nitrosylation and tyrosine nitration [41-46]. Although low nanomolar concentrations of NO donors are sufficient to elicit cGMP-dependent signals, 50-100 μM of NO donors is required for S-nitrosylation-mediated alterations of protein function in cultured cells [37,38,54].

Based on these ideas, we speculate that the NO generated by different NOSs exerts differential modulations of BACE1. For example, the low levels of NO that result in suppression of BACE1 transcription may represent the NO released from vascular eNOS. Although it is difficult to measure the precise concentration of the bioreactive NO in the blood circulation of a healthy vertebrate, the decreased BACE1 transcription induced at the 10-100 nM range of NO donors may be related to the protective actions caused by the release of NO from vascular eNOS. In fact, this is collaborated by the recent finding that BACE1 expression was elevated in mice deficient in eNOS [55]. Our data also suggest that PGC-1α, a crucial PPARγ coactivator in the transcriptional controls of gluconeogenesis and energy metabolism [56], may be the key factor executing the effects of low NO, through the activated cGMP-PKG signaling pathway. Since PGC-1α is the most critical regulator in response to metabolic stress, it is believed to play a key role in AD pathogenesis. Our finding that PGC-1α is likely involved in BACE1 transcriptional control provides the first molecular basis of a metabolic signal/factor regulating an AD gene. In support of this, PGC-1α expression was found to be reduced in AD brains [57] and it was recently reported that PGC-1α facilitates BACE1 protein degradation via the UPS [58,59]. Further characterization of the transcriptional network involving PGC-1α, directly or indirectly, on BACE1 promoter, will draw a fuller picture of the metabolic factors regulating AD genes, which likely involve the entire AMPK-SIRT1-PGC-1α pathway, in which NO signaling plays an important role.

On the other hand, the high levels of NO-mediated BACE1 inactivation via post-translational modification in cultured neurons likely reflects the NO generated by iNOS, known to elicit much higher NO production (over 1,000-fold) compared with that generated by the constitutive NOSs [37,38]. Inducible iNOS, shown to be involved in the pathogenesis of AD, is activated under inflammatory conditions and may upregulate BACE1 as a result of activated NF-κB and toxic peroxynitrite formed by NO and superoxide anions. Although we show that SNO-BACE1 is associated with reduced enzymatic activity, the elevated protein expression and enzymatic activity of BACE1 in late stages of AD may reflect the dominant effect of severe oxidation by H_2_O_2 _and peroxynitrite; it has been shown previously that lipid oxidative products, such as 4-HNE, can upregulate BACE1 transcriptionally [30].

**Figure 6:**
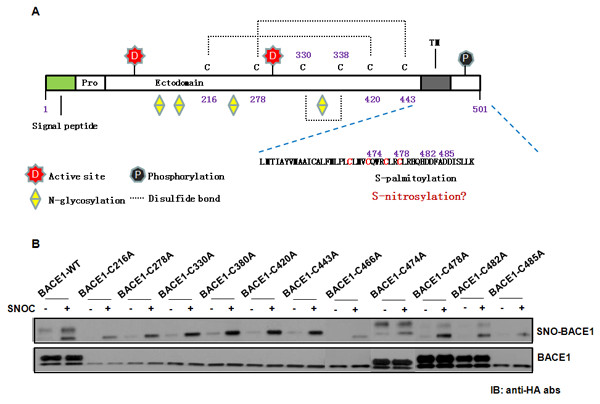
**Mapping crucial cysteine residues for SNO-BACE1**. (A) Schematic diagram of BACE1. BACE1 is a 501 amino-acid transmembrane protein. Signal peptide (1-21 aa) and pro-peptide (22-45 aa) are cleaved during protein maturation. Within the lumenal domain there are two active site motifs (signature sequences of aspartyl proteases) situated at amino acids 93-96 and 289-292, four N-glycosylation sites and six cysteine residues involved in intramolecular disulfide bonds. The transmembrane domain is located between amino acids 461 and 477, and the C-terminal part of the protein (24 aa) is located in the cytosol. (B) Site-directed mutagenesis data on the Cys mutants of BACE1. SNO-BACE1 levels were determined by biotin-switch assays and data were analyzed by densitometry of the SNO-BACE1/total BACE1 ratio to identify crucial cysteine residues for s-nitrosylation of BACE1. Wild type and mutants of BACE1-HA expression plasmids were transfected into HEK293 cells. After biotin-switch assay, SNO-BACE1 and total BACE1 proteins were probed with an anti-HA antibody.

H_2_O_2_-induced modification and S-nitrosylation represent the two dominant oxidative events modifying critical Cys residues in proteins. Interestingly, we observed opposite effects from these two oxidative modifications in BACE1 expression and activity. The interplay between S-nitrosylation and H_2_O_2_-type oxidation of BACE1 at the molecular level is not yet clear, albeit both occur at certain critical Cys residues. Based on our finding that NO supresses BACE1, we speculate that S-nitrosylation and H_2_O_2_-type oxidation occur on overlapping Cys residues on BACE1; nitrosylation precludes further oxidation for enzymatic activation and thus represents a self-defensive or house-keeping mechanism. Among the 11 Cys residues on BACE1, it is known that six Cys residues form three pairs of intramolecular disulfide bonds in mature BACE1 (Cys^216-420^, Cys^278-443 ^and Cys^330-380^; Figure 6A) which is essential for its membrane-association and protein maturation but is not required for its enzymatic activity [60]. Since the pattern of BACE1 S-nitrosylation shows that it occurs predominantly on the mature form of BACE1 (Figure 1A), we reason that the three pairs of Cys in the enzymatic pocket are not likely sites for nitrosylation. Indeed, our preliminary study used site-directed mutagenesis to analyze individual BACE1 mutants with each Cys mutated to Ala and showed that Cys mutation at the positions 216, 278, 330, 380, 420, 443, or 466 (the membrane-proximal ones) resulted in lack of mature BACE1 proteins (Figure 6A). Substitution of each of the four Cys residues in the cytoplasmic tails, which are also the S-palmitoylation sites, had different results; the C483A mutant abolished mature BACE1, making it difficult to assess its contribution as a nitrosylation site; Cys478A and Cys482A mutants appeared to show reduced BACE1 nitrosylation and likely represent S-nitrosylation sites under physiological conditions. It should be pointed out that the results of this type of semi-quantitative analysis are not sufficient to determine the molecular basis of nitrosylation sites unambiguously. In particular, the cytoplasmic tail has been reported to be non-essential for the enzymatic activity of BACE1 [61,62], it remains unclear how the nitrosylation affects BACE1 activity. Additional research to determine the molecular basis of the nitrosylation and oxidation events by quantitative mass spectrometry, as well as further analysis of BACE1 and subcellular trafficking, localization and distribution in lipid rafts using cell biology approaches, are necessary to clarify the mechanism.

## Conclusion

In summary, our studies have demonstrated that NO exerts differential regulation on BACE1 at low and modest to high concentrations, suppressing BACE1 transcriptionally or post-translationally through S-nitrosylation, respectively. S-nitrosylation may represent a basic regulatory mechanism for maintaining BACE1 at physiological levels, outside of events that challenge the brain with a wave of high oxidative stressors which upregulates and activates BACE1. As BACE1 represents a favored therapeutic target for developing anti-AD agents, pharmacological inhibitors of BACE1 have been actively pursued for more than a decade. Despite significant progress, the development of specific cell-permeable drugs that penetrate into the brain remains a challenge. Due to the recent discouraging results from Eli Lilly's trials on a γ-secretase inhibitor (Semagacestat/LY450139) showing worsening cognitive performance in AD patients, efforts to discover a novel mechanism to modulate secretases is of particular importance. Further validation of the redox regulatory mechanism of BACE1 may provide novel leads to modulate BACE1 by selectively targeting oxidized BACE1.

## Methods

### Cell culture, treatment of neurotoxic reagents and transfection

Primary cultured cortical neurons (PRCN), neuroblastoma N2a cells and HEK 293 cells were prepared as described [63]. N2a cells stably transfected with human APP695 (N2a695 cells) were maintained in N2a culture media supplemented with 400 μg/mL G418 (Sigma). For the majority of experiments, freshly prepared SNOC (Sigma-Aldrich, St. Louis, MO) was added at 100-200 μM to these cells for 30 or 60 min. SNOC was freshly prepared as described [54]. In brief, to prepare a 100 mM stock solution, 0.0069 g sodium nitrite and 0.0121 g l-cysteine were added to 950 μl H_2_O. Then, 50 μl of 10N HCl is added to adjust pH to 7.4. For Aβ_25-35 _peptides (Bachem, Torrance, CA), neurons were treated for 4 hours before cell lysates were prepared. Transient transfections were performed with plasmid constructs for pCMV-HA-BACE1-WT and its site-directed mutagenized constructs (Cysteine to Alanine substitution) according to manufacturer's protocol.

### Human patient brains

Human brain samples were provided by UCSD, San Diego, CA and were analyzed with institutional permission under California and National Institutes of Health guidelines [Additional file 1]. Informed consent was obtained following the procedures of the Institutional Review Boards of the Sanford-Burnham Institute for Medical Research.

### Biotin-switch assay for detection of BACE1 S-nitrosylation

A biotin switch assay for detection of SNO-BACE1 was performed as previously described with minor modifications [48]. PRCN, N2a or HEK293 cells were exposed to various concentrations (0.01, 0.1, 10, 50, 100, 200 and 400 μM) of SNOC for 30 min. For calcium ionophore (ionomycin), 1 μM of ionomycin was applied for 0-5-10-15-30 min to neuronal cells.

### Western blot analysis

SDS-PAGE gels and Western blots were performed as described [64]. Samples were run on 10-20% Tricine gels (Invitrogen) for Aβ peptides and 4-20% Tris-Glycine gels (Invitrogen) for regular SDS-PAGE analysis. The following antibodies (Abs) were used in the present study: mouse anti-HA Ab (Sigma); mouse anti-α-tubulin Ab (Sigma, St. Louis, MO, USA); mouse anti-β-actin Ab (Sigma); anti-mouse IgG and anti-rabbit IgG horseradish peroxidase-conjugated Abs (Chemicon, Temecula, CA, USA). In most of the experiments performed in the early stages, we used the guinea pig antibody GP45 (Convance, Inc). Recently, we repeated some key experiments with a specific monoclonal antibody (5D3) derived from BACE1 deficient mice [9].

### Fluorogenic BACE 1 activity assay

This assay was performed according to the company's protocol [64]. Briefly, cultured PRCN, N2a and HEK293 cells were treated with or without SNOC and H_2_O_2 _for the indicated time periods and homogenized. The resulting aliquots (containing 15 μg of proteins) were centrifuged at 13 000 × g for 15 min. Then, the membrane pellets were recovered and incubated at 37°C for 30 min in 50 μl of assay reaction buffer (50 mM sodium acetate, pH 4.5; for α-secretase, 10 mM Tris-HCl, pH 7.5) containing 10 μM specific fluorogenic substrates. After incubation, fluorescence was measured using a spectrometer at excitation/emission wavelengths of 320/420 nm for BACE-1.

### Aβ assays

Following various treatments, serum-free media was conditioned for 4 hrs. Aliquots of conditioned media were immunoprecipiated with 6E10 antibody and protein G/A beads to measure Aβ peptides [65].

### Quantitative RT-PCR on BACE1 and PGC-1 Messages

Total RNA was extracted using TRIzol reagent (Invitrogen). SuperScript First-Strand kit (Invitrogen) was used to synthesize the first strand cDNA from samples with an equal amount of RNA, according to the manufacturer's instructions. Synthesized cDNAs were amplified using RealMasterMix SYBR ROX (5 Prime) and Mastercycler ep from Eppendorf with 3 min pre-incubation at 95°C followed by 40 cycles of 30 s at 95°C, 30 s at 55°C, 30 s at 72°C.;. The data were analyzed by using realplex. PCR products were verified by melting curve analysis and agarose gel electrophoresis. Rat bace1 primers [forward 5'-TGGGTGAAGTCACCAATCAG-3' and reverse 5'-CACTGGCCGTAGGTATTGCT-3'], human bace1 primers [ forward 5'-GCAGGGCTACTACGTGGAGA-3' and reverse 5'-GTATCCACCAGGATGTTGAGC-3'], and rat pgc-1α primers [forward 5'-AAAGGGCCAAGCAGAGAGA-3' and reverse 5'-GTAAATCACACGGCGCTCTT-3'] were used in the present studies. Primers used for rat GAPDH, forward 5'-ACATTGTTGCCATCAACGAC-3', reverse 5'-CTTGCCGTGGGTAGCGTCAT-3'; human GAPDH primers, forward 5'-AATCCCATCACCATCTTCC-3' and reverse 5'-GGACTCCACGACGTACTCA-3'. BACE1 and PGC-1 alpha mRNA levels were normalized with the levels of GAPDH.

### Luciferase assay for BACE1 promoter activity

1.5 Kb rat BACE1 promoter [63] and 2.2 Kb human BACE1 promoter [19] in pGL3-Basic vector, together with PGC-1α expression vector (Addgene), were transfected into HEK 293 cells. pRL-SV40 containing the Renilla luciferase gene (Promega) was cotransfected as an internal control. 24 hours after transfection, cells were collected in passive lysis buffer and analyzed with the Dual luciferase reporter system (Promega) following the manufacturer's instructions.

### Site-directed mutagenesis of BACE1

BACE1 mutants were created by site-directed mutagenesis (QuikChange^® ^II Site-Directed Mutagenesis Kits, Stratagene, La Jolla USA) at C216, C278, C330, C380, C420, C443, C466, C474, C478, C482, and C485 residues. The primers for C216A forward 5'-CTCCCTGCAGCTTgctGGTGCTGGCTTCCC-3' and reverse 5'-GGGAAGCCAGCACCagcAAGCTGCAGGGAG-3'; C278A forward 5'-CTGAAAATGGACgccAAGGAGTACAAC-3' and reverse 5'-GTTGTACTCCTTggcGTCCATTTTCAG-3'; C330A forward 5'-GAGCAGCTGGTGgccTGGCAAGCAGGC-3' and reverse 5'-GCCTGCTTGCCAggcCACCAGCTGCTC-3'; C380A forward 5'-GTCCCAAGACGACgctTACAAGTTTGCC-3' and reverse 5'-GGCAAACTTGTAagcGTCGTCTTGGGAC-3'; C420A forward 5'-GCTGTCAGCGCTgccCATGTGCACGATG-3' and reverse 5'-CATCGTGCACATGggcAGCGCTGACAGC-3'; C443A forward 5'-GACATGGAAGACgctGGCTACAACATTC-3' and reverse 5'-GAATGTTGTAGCCagcGTCTTCCATGTC-3'; C466A forward 5'-CATGGCTGCCATCgccGCCCTCTTCATG-3' and reverse 5'-CATGAAGAGGGCggcGATGGCAGCCATG-3'; C474A forward 5'-CATGCTGCCACTCgccCTCATGGTGTGTC-3' and reverse 5'-CTGCTGGCGCAGggcGCGGAGGCAGCGC-3'; were utilized for PCR amplification. PCR amplification was performed according to the company's protocol.

### Statistics

All quantitative data were presented as means ± SDV. Comparisons between groups were analyzed with unpaired ANOVA using Graphpad PRIZM software (La Jolla, CA, USA) and values of *p *< 0.05 were considered to be significant.

## Competing interests

The authors declare that they have no competing interests.

## Authors' contributions

Author contributions: Y-DK and FFL designed research; Y-DK, RW, and J-JL performed experiments; Y-W Z, HX, analyzed data. FFL and Y-DK wrote the paper. All authors have read and approved the final manuscript.

## Supplementary Material

Additional file 1 **The table of the patient cohort and information**. Click here for file

## References

[B1] Cechetto DF Hachinski V Whitehead SN Vascular risk factors and Alzheimer's disease Expert Rev Neurother 2008 8 743 750 Review 10.1586/14737175.8.5.743 18457531

[B2] Purnell C Gao S Callahan CM Hendrie HC Cardiovascular risk factors and incident Alzheimer disease: a systematic review of the literature Alzheimer Dis Assoc Disord 2009 23 1 10 Review 10.1097/WAD.0b013e318187541c 18703981 PMC3689425

[B3] Janson J Laedtke T Parisi JE O'Brien P Petersen RC Butler PC Increased risk of type 2 diabetes in Alzheimer disease Diabetes 2004 53 474 481 10.2337/diabetes.53.2.474 14747300

[B4] Biessels GJ Kappelle LJ Increased risk of Alzheimer's disease in Type II diabetes: insulin resistance of the brain or insulin-induced amyloid pathology? Biochem Soc Trans 2005 33 1041 1044 10.1042/BST20051041 16246041

[B5] Reddy VP Zhu X Perry G Smith MA Oxidative stress in diabetes and Alzheimer's disease J Alzheimers Dis 2009 6 763 774 Review 10.3233/JAD-2009-1013 PMC2765716 19387111

[B6] Lovell MA Markesbery WR Oxidative DNA damage in mild cognitive impairment and late-stage Alzheimer's disease Nucleic Acids Res 2007 35 7497 7504 10.1093/nar/gkm821 17947327 PMC2190704

[B7] Sultana R Perluigi M Butterfield DA Oxidatively modified proteins in Alzheimer's disease (AD), mild cognitive impairment and animal models of AD: role of Abeta in pathogenesis Acta Neuropathol 2009 18 131 150 Review 10.1007/s00401-009-0517-0 PMC2818870 19288120

[B8] Vetrivel KS Zhang YW Xu H Thinakaran G Pathological and physiological functions of presenilins Mol Neurodegener 2006 1 4 10.1186/1750-1326-1-4 16930451 PMC1513131

[B9] Cole SL Vassar R The Alzheimer's disease beta-secretase enzyme, BACE1 Mol Neurodegener 2007 2 22 10.1186/1750-1326-2-22 18005427 PMC2211305

[B10] Zhang YW Xu H Molecular and cellular mechanisms for Alzheimer's disease: understanding APP metabolism Curr Mol Med 2007 7 687 696 Review 10.2174/156652407782564462 18045146

[B11] Fukumoto H Cheung BS Hyman BT Irizarry MC Beta-secretase protein and activity are increased in the neocortex in Alzheimer disease Arch Neurol 2002 59 1381 1389 10.1001/archneur.59.9.1381 12223024

[B12] Tyler SJ Dawbarn D Wilcock GK Allen SJ alpha- and beta-secretase: profound changes in Alzheimer disease Biochem Biophys Res Comm 2002 299 373 376 10.1016/S0006-291X(02)02635-9 12445809

[B13] Holsinger RM McLean CA Beyreuther K Masters CL Evin G Increased expression of the amyloid precursor beta-secretase in Alzheimer's disease Ann Neurol 2002 51 783 786 10.1002/ana.10208 12112088

[B14] Yang LB Lindholm K Yan R Citron M Xia W Yang XL Beach T Sue L Wong P Price D Li R Shen Y Elevated beta-secretase expression and enzymatic activity detected in sporadic Alzheimer disease Nat Med 2003 9 3 4 10.1038/nm0103-3 12514700

[B15] Li R Linholm K Yang LB Yue X Citron M Yan R Beach T Sue L Sabbagh M Cai H Wong P Price D Shen Y Amyloid beta peptide load is correlated with increased beta-secretase activity in sporadic Alzheimer's disease patients Proc Natl Acad Sci USA 2004 101 3632 3637 10.1073/pnas.0205689101 14978286 PMC373514

[B16] Luo Y Bolon B Kahn S Bennett BD Babu-Khan Sm Denis P Pan W Kha H Zhang J Gong Y Martin L Louis JC Yan Q Richards WG Citron M Vassar R Mice deficient in BACE1, the Alzheimer's b-secretase, have normal phenotype and abolished b-amyloid generation Nature Neurosci 2001 4 231 232 10.1038/85059 11224535

[B17] Roberds SL Anderson J Basi G Bienkowski MJ Branstetter DG Chen KS Freedman SB Frigon NL Games D Hu K Johnson-Wood K Kappenman KE Kawabe TT Kola I Kuehn R Lee M Liu W Motter R Nichols NF Power M Robertson DW Schenk D Schoor M Shopp GM Shuck ME Sinha S Svensson KA Tatsuno G Tintrup H Wijsman J Wright S McConlogue L BACE knockout mice are healthy sespite lacking the primary b-secretase activity in brain: implications for Alzheimer's disease therapeutics Hum Mol Genet 2001 10 1317 1324 10.1093/hmg/10.12.1317 11406613

[B18] Lammich S Schobel S Zimmer AK Lichtenthaler SF Haass C Expression of the Alzheimer protease BACE1 is suppressed via its 5'-untranslated region EMBO Rep 2004 5 620 625 10.1038/sj.embor.7400166 15167888 PMC1299076

[B19] Rossner S Sastre M Bourne K Lichtenthaler SF Transcriptional and translational regulation of BACE1 expression-implications for Alzhemeri's disease Prog Neurobiol 2006 79 95 111 10.1016/j.pneurobio.2006.06.001 16904810

[B20] Christensen MA Zhou W Qing H Lehman A Philipsen S Song W Transcriptional regulation of BACE1, the beta-amyloid precursor protein beta-secretase, by Sp1 Mol cell Biol 2004 24 865 874 10.1128/MCB.24.2.865-874.2004 14701757 PMC343820

[B21] Sun X Wang Y Qing H Christensen MA Liu Y Zhou W Tong Y Xiao C Huang Y Zhang S Liu X Song W Distinct transcriptional regulation and function of the human BACE2 and BACE1 genes FASEB J 2005 19 739 749 10.1096/fj.04-3426com 15857888

[B22] Ge YW Maloney B Sambamurti K Lahiri DK Functional characterization of the 5' flanking region of the BACE gene: identification of a 91 bp fragment involved in basal level of BACE promoter expression FASEB J 2004 18 1037 1039 15059977 10.1096/fj.03-1379fje

[B23] Wang WX Rajeev BW Stromberg AJ Ren N Tang G Huang Q Rigoutsos I Nelson PT The expression of microRNA miR-107 decreases early in Alzheimer's disease and may accelerate disease progression through regulation of beta-site amyloid precursor protein-cleaving enzyme 1 J Neurosci 2008 28 1213 1223 10.1523/JNEUROSCI.5065-07.2008 18234899 PMC2837363

[B24] Hébert SS Horré K Nicolaï L Papadopoulou AS Mandemakers W Silahtaroglu AN Kauppinen S Delacourte A De Strooper B Loss of microRNA cluster miR-29a/b-1 in sporadic Alzheimer's disease correlates with increased BACE1/beta-secretase expression Proc Natl Acad Sci USA 2008 105 6415 6420 18434550 10.1073/pnas.0710263105 PMC2359789

[B25] Nelson PT Wang WX MiR-107 is reduced in Alzheimer's disease brain neocortex: validation study J Alzheimers Dis 2010 21 75 79 20413881 10.3233/JAD-2010-091603 PMC2910235

[B26] Blasko I Beer R Bigl M Apelt J Franz G Rudzki D Ransmayr G Kampfl A Schliebs R Experimental traumatic brain injury in rats stimulates the expression, production and activity of Alzheimer's disease beta-secretase (BACE-1) J Neural Transm 2004 111 523 536 10.1007/s00702-003-0095-6 15057522

[B27] Wen Y Onyewuchi O Yang S Liu R Simpkins JW Increased beta-secretase activity and expression in rats following transient cerebral ischemia Brain Res 2004 1009 1 8 10.1016/j.brainres.2003.09.086 15120577

[B28] Tesco G Koh YH Kang EL Cameron AN Das S Sena-Esteves M Hiltunen M Yang SH Zhong Z Shen Y Simpkins JW Tanzi RE Depletion of GGA3 stabilizes BACE and enhances beta-secretase activity Neuron 2007 54 721 737 10.1016/j.neuron.2007.05.012 17553422 PMC1973166

[B29] Tamagno E Bardini P Obbili A Vitali A Borghi R Zaccheo D Pronzato MA Danni O Smith MA Perry G Tabaton M Oxidative stress increases expression and activity of BACE in NT2 neurons Neurobiol Dis 2002 10 279 288 10.1006/nbdi.2002.0515 12270690

[B30] Tamagno E Parola M Bardini P Piccini A Borghi R Guglielmotto M Santoro G Davit A Danni O Smith MA Perry G Tabaton M Beta-site APP cleaving enzyme up-regulation induced by 4-hydroxynonenal is mediated by stress-activated protein kinases pathways J Neurochem 2005 92 628 636 10.1111/j.1471-4159.2004.02895.x 15659232

[B31] Chen L Na R Gu M Richardson A Ran Q Lipid peroxidation up-regulates BACE1 expression in vivo: a possible early event of amyloidogenesis in Alzheimer's disease J Neurochem 2008 107 197 207 10.1111/j.1471-4159.2008.05603.x 18680556 PMC2716044

[B32] Sun X He G Qing H Zhou W Dobie F Cai F Staufenbiel M Huang LE Song W Hypoxia facilitates Alzheimer's disease pathogenesis by up-regulating BACE1 gene expression Proc Natl Acad Sci USA 2006 103 18727 18732 10.1073/pnas.0606298103 17121991 PMC1693730

[B33] Zhang X Zhou K wang R Cui K Lipton SA Liao FF Xu H Zhang YW Hypoxia-inducible factor 1alpha (Hif-1alpha)-mediated hypoxia increases BACE1 expression and beta-amyloid generation J Biol Chem 2007 282 10873 10880 10.1074/jbc.M608856200 17303576

[B34] Guglielmotto M Aragno M Autelli R Giliberto L Novo E Colombatto S Danni O Parola M Smith MA Perry G Tamagno E Tabaton M The up-regulation of BACE1 mediated by hypoxia and ischemic injury: role of oxidative stress and HIF1alpha J Neurochem 2009 108 1045 1056 10.1111/j.1471-4159.2008.05858.x 19196431

[B35] Bredt DS Snyder SH Nitric oxide: a physiologic messenger molecule Annu Rev Biochem 1994 63 175 195 Review 10.1146/annurev.bi.63.070194.001135 7526779

[B36] Kuo PC Schroeder RA The emerging multifaceted roles of nitric oxide Ann Surg 1995 221 220 223 10.1097/00000658-199503000-00003 7717775 PMC1234563

[B37] Drew B Leeuwenburgh C Aging and the role of reactive nitrogen species Ann N Y Acad Sci 2002 959 66 81 10.1111/j.1749-6632.2002.tb02084.x 11976187

[B38] Wink DA Miranda KM Espey MG Effects of oxidative and nitrosative stress in cytotoxicity Semin Perinatol 2000 24 20 23 10.1016/S0146-0005(00)80049-X 10709853

[B39] Calabrese V Cornelius C Rizzarelli E Owen JB Dinkova-Kostova AT Butterfield DA Nitric oxide in cell survival: a janus molecule Antioxid Redox Signal 2009 11 2717 2739 10.1089/ars.2009.2721 19558211

[B40] Smith MA Zhu X Tabaton M Liu G McKeel DW Jr Cohen ML Wang X Siedlak SL Dwyer BE Hayashi T Nakamura M Nunomura A Perry G Increased iron and free radical generation in preclinical Alzheimer disease and mild cognitive impairment J Alzheimers Dis 2010 19 363 337 20061651 10.3233/JAD-2010-1239 PMC2842004

[B41] Lipton SA Singel DJ Stamler JS Nitric oxide in the central nervous system Prog Brain Res 1994 103 359 364 full_text 7886218 10.1016/s0079-6123(08)61149-8

[B42] Stamler JS Toone EJ Lipton SA Sucher NJ (S)NO Signals; translocation, regulation and a concensus motif Neuron 1997 18 691 695 10.1016/S0896-6273(00)80310-4 9182795

[B43] Dawson VL Sawson TM Nitric oxide in neurodegeneration Prog Brain Res 1998 118 215 229 full_text 9932444 10.1016/s0079-6123(08)63210-0

[B44] Foster MW Hess DT Stamler JS Protein S-nitrosylation in health and disease: a current perspective Trends Mol Med 2009 15 391 404 10.1016/j.molmed.2009.06.007 19726230 PMC3106339

[B45] Nakamura T Lipton SA Cell death: protein misfolding and neurodegenerative diseases Apoptosis 2009 14 455 468 Review 10.1007/s10495-008-0301-y 19130231 PMC13339731

[B46] Gu Z Nakamura T Lipton SA Redox reactions induced by nitrosative stress mediate protein misfolding and mitochondrial dysfunction in neurodegenerative diseases Mol Neurobiol 2010 41 55 72 10.1007/s12035-010-8113-9 20333559 PMC4586261

[B47] Kwak YD Ma T Diao S Zhang X Chen Y Hsu J Lipton SA Masliah E Xu H Liao FF NO signaling and S-nitrosylation regulate PTEN inhibition in neurodegeneration Mol Neurodegener 2010 5 49 10.1186/1750-1326-5-49 21067594 PMC2992530

[B48] Jaffrey SR Synder SH The biotin switch method for the detection of S-nitrosylated proteins Sci STKE 2001 2001 11 10.1126/stke.2001.86.pl1 11752655

[B49] Haendeler J Hoffmann J Tischler V Berk BC Zeiher AM Dimmeler S Redox regulatory and anti-apoptotic functions of thioredoxin depend on Snitrosylation at cysteine 69 Nat Cell Biol 2002 4 743 774 10.1038/ncb851 12244325

[B50] Borghi R Patriarca S Traverso N Piccini A Storace D Garuti A Cirmena Gabriella Odetti Patrizio Tabaton Massimo The increased activity of BACE1 correlates with oxidative stress in Alzheimer's disease Neurobiol Aging 2007 28 1009 1014 10.1016/j.neurobiolaging.2006.05.004 16769154

[B51] Tong Y Zhou W Fung V Christensen MA Qing H Sun X Song W Oxidative stress potentiates BACE1 gene expression and Aβ generation J Neural Transm 2005 112 455 469 10.1007/s00702-004-0255-3 15614428

[B52] Nisoli E Clementi E Paolucci C Cozzi V Tonello C Sciorati C Bracale R Valerio A Francolini M Moncada S Carruba MO Mitochondrial biogenesis in mammals: the role of endogenous nitric oxide Science 2003 299 896 899 10.1126/science.1079368 12574632

[B53] Sastre M Dewachter I Rossner S Bogdanovic N Rosen E Borghgraef P Evert BO Dumitrescu-Ozimek L Thal DR Landreth G Walter J Klockgether T van Leuven F Heneka MT Nonsteroidal anti-inflammatory drugs repress beta-secretase gene promoter activity by the activation of PPARgamma Proc Natl Acad Sci USA 2006 103 443 448 10.1073/pnas.0503839103 16407166 PMC1326151

[B54] Lei SZ Pan ZH Aggarwal SK Chen HS Hartman J Sucher NJ Lipton SA Effect of nitric *oxide production on the redox modulatory site of the NMDA receptor-channel complex* Neuron 1992 8 1087 1099 10.1016/0896-6273(92)90130-6 1376999

[B55] Austin SA Santhanam AV Katusic ZS Endothelial nitric oxide modulates expression and porocessing of amyloid precursor protein Circ Res 2010 107 1498 1502 10.1161/CIRCRESAHA.110.233080 21127294 PMC3064266

[B56] Lin J Handschin C Spiegelman BM Metabolic control through the PGC-1 family of transcription coactivators Cell Metab 2005 1 361 370 10.1016/j.cmet.2005.05.004 16054085

[B57] Qin W Haroutunian V Katsel P Cardozo CP Ho L Buxbaum JD Pasinetti GM PGC-1 expression decreases in the Alzheimer disease brain as a function of dementia Arch Neurol 2009 66 352 361 10.1001/archneurol.2008.588 19273754 PMC3052997

[B58] Qing H Zhou W Christensen MA Sun X Tong Y Song W Degradation of BACE by the ubiquitin-proteasome pathway FASEB J 2004 18 17141 17149 10.1096/fj.04-1994fje 15289451

[B59] Gong B Chen F Pan Y Arrieta-Cruz I Yoshida Y Haroutunian V Pasinetti GM SCFFbx2-E3-ligase-mediated degradation of BACE1 attenuates Alzheimer's disease amyloidosis and improves synaptic function Aging Cell 2010 9 1018 1031 10.1111/j.1474-9726.2010.00632.x 20854419 PMC3307224

[B60] Fischer F Molinari M Bodendorf U Paganetti P The disulphide bonds in the catalytic domain of BACE are critical but not essential for amyloid precursor protein processing activity J Neurochem 2002 80 1079 1088 10.1046/j.0022-3042.2002.00806.x 11953458

[B61] Pastorino L Ikin AF Nairn AC Pursnani A Buxbaum JD The carboxyl-terminus of BACE contains a sorting signal that regulates BACE trafficking but not the formation of total A{beta} Mol Cell Neurosci 2002 19 175 185 10.1006/mcne.2001.1065 11860271

[B62] Vetrivel KS Meckler X Chen Y Nguyen P Seidah NG Vassar R Wong PC Fukata M Kounnas MZ Thinakaran G Alzheimer disease Aβ production in the absence of S-palmitoy lation-dependent targeting of BACE1 to lipid rafts J Biol Chem 2009 284 3793 3803 10.1074/jbc.M808920200 19074428 PMC2635050

[B63] Ma T Zhao Y Kwak YD Yang Z Thompson R Luo Z Xu H Liao FF Statin's excitoprotection is mediated by sAPP and the subsequent attenuation of calpain-induced truncation events, likely via rho-ROCK signaling J Neurosci 2009 29 11226 11236 10.1523/JNEUROSCI.6150-08.2009 19741129 PMC2757921

[B64] Chen Y Zhou K Wang R Liu Y Kwak YD Ma T Thompson RC Zhao Y Smith L Gasparini L Luo Z Xu H Liao FF Antidiabetic drug metformin (GlucophageR) increases biogenesis of Alzheimer's amyloid peptides via up-regulating BACE1 transcription Proc Natl Acad Sci USA 2009 106 3907 3912 10.1073/pnas.0807991106 19237574 PMC2656178

[B65] Huang X Chen Y Li WB Cohen SN Liao FF Li L Xu H Zhang YW The Rps23rg gene family originated through retroposition of the ribosomal protein s23 mRNA and encodes proteins that decrease Alzheimer's beta-amyloid level and tau phosphorylation Hum Mol Genet 2010 19 3835 3843 10.1093/hmg/ddq302 20650958 PMC2935860

