# Supplementary figures and images for: Differential regulation of BACE1 expression by oxidative and nitrosative signals
Source: Mol Neurodegener. 2011 Mar 3;6:17. doi: 10.1186/1750-1326-6-17 (PMC3059281; doi:10.1186/1750-1326-6-17)

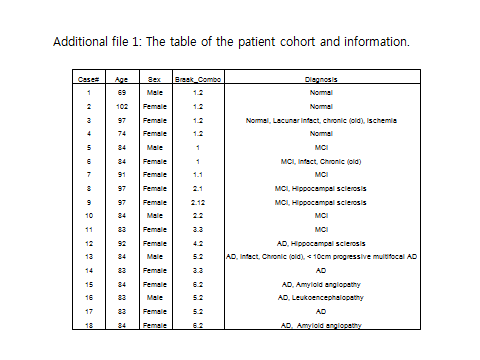

Supplement: Additional file 1 — The table of the patient cohort and information. [file 1750-1326-6-17-S1.TIFF]
